# Cognitive and Emotional Alterations Are Related to Hippocampal Inflammation in a Mouse Model of Metabolic Syndrome

**DOI:** 10.1371/journal.pone.0024325

**Published:** 2011-09-16

**Authors:** Anne-Laure Dinel, Caroline André, Agnès Aubert, Guillaume Ferreira, Sophie Layé, Nathalie Castanon

**Affiliations:** 1 Nutrition et Neurobiologie Intégrée, INRA UMR 1286, Bordeaux, France; 2 University of Bordeaux, Bordeaux, France; Virginia Commonwealth University, United States of America

## Abstract

Converging clinical data suggest that peripheral inflammation is likely involved in the pathogenesis of the neuropsychiatric symptoms associated with metabolic syndrome (MetS). However, the question arises as to whether the increased prevalence of behavioral alterations in MetS is also associated with central inflammation, i.e. cytokine activation, in brain areas particularly involved in controlling behavior. To answer this question, we measured in a mouse model of MetS, namely the diabetic and obese *db/db* mice, and in their healthy *db/+* littermates emotional behaviors and memory performances, as well as plasma levels and brain expression (hippocampus; hypothalamus) of inflammatory cytokines. Our results shows that *db/db* mice displayed increased anxiety-like behaviors in the open-field and the elevated plus-maze (i.e. reduced percent of time spent in anxiogenic areas of each device), but not depressive-like behaviors as assessed by immobility time in the forced swim and tail suspension tests. Moreover, *db/db* mice displayed impaired spatial recognition memory (hippocampus-dependent task), but unaltered object recognition memory (hippocampus-independent task). In agreement with the well-established role of the hippocampus in anxiety-like behavior and spatial memory, behavioral alterations of *db/db* mice were associated with increased inflammatory cytokines (interleukin-1β, tumor necrosis factor-α and interleukin-6) and reduced expression of brain-derived neurotrophic factor (BDNF) in the hippocampus but not the hypothalamus. These results strongly point to interactions between cytokines and central processes involving the hippocampus as important contributing factor to the behavioral alterations of *db/db* mice. These findings may prove valuable for introducing novel approaches to treat neuropsychiatric complications associated with MetS.

## Introduction

For several decades, the prevalence of the metabolic syndrome (MetS) and related comorbidities continuously increases worldwide at alarming rate. This syndrome is defined as a constellation of interrelated metabolic dysregulations, including abdominal obesity, hypertension, hyperglycemia, insulin-resistance, leptin-resistance and hypercortisolemia [1]–[3].

Mounting evidence highlights that patients with MetS often experience a higher prevalence of mood symptoms and cognitive dysfunctions than the general age-matched population [4]–[6]. The overwhelming influence of metabolic and neuropsychiatric disorders considerably impairs the quality of life of MetS patients and results in incremental costs to health care systems around the world [7]. Actually, neuropsychiatric symptoms emerge as significant risk factors for aggravation of MetS and related health outcomes, particularly cardiovascular diseases and type 2 diabetes (T2D) [8]–[12]. Even so, relatively little is known about the pathophysiological mechanisms contributing to the development of neuropsychiatric symptoms in the context of MetS. Recent evidence suggests that some major biological systems, including the inflammatory system, may participate in both MetS and neuropsychiatric disorders [13]–[15].

Inflammation is a key component of MetS, as elevated circulating levels of inflammatory mediators facilitate the development of this condition [16], [17]. Severe obesity is associated with an inflammatory profile characterized by increased plasma production of cytokines and resulting in an imbalance of the cytokine network [18]. Similarly, T2D is associated with a shift of the balance between proinflammatory and anti-inflammatory cytokines toward inflammation [19]. Consequently, MetS is presently viewed not only as a metabolic disorder, but also as an inflammatory disease affecting both innate and acquired immune systems [17], [20].

Abundant evidence supports immune-to-brain communication, with peripheral cytokines acting on the brain to induce local production of cytokines and to influence pathways involved in the regulation of mood and cognition, including neurotransmitter metabolism, neuroendocrine function and neural plasticity [21], [22]. Interestingly, we and others have identified peripheral and central inflammatory factors as important mediators of the neuropsychiatric symptoms observed in many medical illnesses sharing chronic systemic inflammation as a common denominator [22]–[26]. We have found in mice that the selective activation of enzymatic pathways involved in brain monoamine metabolism by interferon-γ (IFN-γ) and tumor necrosis factor-α (TNF-α) mediates development of depressive-like behaviors in response to chronic infection [24]–[26]. The same mechanisms likely contribute to the high incidence of depressive disorders reported in medically ill patients chronically treated with IFN-α [27] or in elderly subjects exhibiting systemic low-grade inflammation as manifested by increased serum levels of C-reactive protein (CRP) and interleukin-6 (IL-6) [28]. Besides, converging animal [29]–[31] and clinical findings [32]–[34] support a main role for IL-6 in mood disorders and cognitive decline. Mounting evidence suggests that systemic inflammation, particularly higher peripheral IL-6, is associated with brain inflammation [35], [36] that might adversely affect mood, learning and memory through processes related to neurodegeneration and structural remodeling [37]–[39]. These processes mainly affect the hippocampus [40]–[42], a key brain area for memory formation and mood [43], [44].

A sizeable number of molecules are well-known to influence hippocampal synaptic plasticity contributing to mood and memory processes, but the brain-derived neurotrophic factor (BDNF) is a major candidate in the context of MetS. BDNF has been found critical in a number of memory tasks [45] and represents an essential constituent of central neural circuits involved in regulating energy homeostasis [46]. Intact hippocampal BDNF signaling determines antidepressant efficacy and influences anxiety-like behaviors [47]. Moreover, hippocampal BDNF mRNA expression and signal transduction are negatively regulated by proinflammatory cytokines [48]–[50], with reported consequences on cytokine-induced behavioral alterations [21], [48], [49].

There are but few reports concerned with the role of inflammation in the onset of neuropsychiatric symptoms in patients with MetS and/or obesity. Increased brain levels of TNF-α and IL-6 induced by consumption of high-fat diet in mice are associated with reduced BDNF levels and cognitive performances [51]. At the clinical levels, cognitive impairments have been recently reported to be more likely associated with MetS in the presence of elevated circulating levels of CRP [11], [52]. Other clinical reports point to elevated circulating levels of IL-6 as important determinant of mood symptoms [53], [54] and cognitive decline [32] associated with MetS and/or obesity. In light of the present knowledge on the neurobiological and behavioral consequences of elevated IL-6 concentrations, these data strongly suggest that brain inflammation is likely involved in the pathogenesis of the neuropsychiatric symptoms associated with MetS. However, clinical studies in patients with MetS only assess systemic inflammation and it is still unknown if enhanced cytokine production associated with neuropsychiatric disorders in MetS does exist in brain areas involved in controlling behavior, particularly the hippocampus. In this context, corollary questions arise as to whether all neuropsychiatric symptoms are equally affected in MetS and which inflammatory pathways are potentially activated in the hippocampus.

In the present study, we sought to answer these questions in a mouse model of MetS, the *db/db* mice, which display T2D, obesity, hyperglycemia, insulin-resistance and hyperinsulinemia as a consequence of an inactivating mutation in the leptin receptor [55]. There are but few reports showing that *db/db* mice also display immune changes, particularly altered immune response to infection both in terms of cytokine production [56], [57] and sickness behavior [58]. Few studies have dealt with the emotional behaviors and cognitive performances of *db/db* mice [59]–[62], but there are as yet no report regarding the potential involvement of inflammatory processes, particularly within the brain, in impaired emotional and cognitive behaviors. Therefore, the present set of experiments was carried out to identify in *db/db* mice potential alterations in depressive-like, anxiety-like and cognitive behaviors together with a detailed assessment of their peripheral and central inflammatory status. According to the role of the hippocampus in emotional and cognitive behaviors [43], [44] we hypothesized that the behavioral deficits of *db/db* mice would be specifically associated with activation of cytokine pathways in the hippocampus.

## Results

### 1–*db/db* mice display expected metabolic dysregulations

In accordance with their expected phenotype, *db/db* mice were markedly heavier than *db/+* controls (weight (g): 25.5±0.3 *vs.* 40.5±1.0 at 10 week-old; F_(1,26)_ = 220.0, p<0.0001), ate more (daily food intake (g): 4.5±0.2 *vs.* 7.8±0.5 during the last week before sacrifice; F_(1,25)_ = 43.6, p<0.0001) and showed a marked increase in the proportion of adipose tissue compared to *db/+* mice (data not shown). This was associated with hyperleptinemia (F_(1,10)_ = 266.2, p<0.0001), hyperinsulinemia (F_(1,10)_ = 21.1, p<0.001), hyperglycemia (F_(1,8)_ = 14.3, p<0.005) and hypercortisolemia (F_(1,10)_ = 11.4, p<0.01), but reduced plasma concentrations of resistin (F_(1,10)_ = 13.4, p<0.005) (Table 1).

**Table 1:** Concentrations of metabolic hormones and cytokines in the plasma of *db/+* and *db/db* mice.

Plasma	*db/+*	*db/db*
**Leptin (ng/ml)**	2.8±0.7	63.7±3.7***
**Resistin (ng/ml)**	3.4±0.1	1.7±0.1**
**Insulin (ng/ml)**	1.1±0.2	5.9±1.0***
**Glucose (mg/dl)**	220.2±14.7	632.8±135.5**
**Corticosterone (ng/ml)**	26.2±6.1	148.2±38.61**
**IL-1β (pg/ml)**	17.4±9.6	29.0±11.5
**TNF-α (pg/ml)**	8.7±1.1	13.9±5.1
**IL-6 (pg/ml)**	11.7±5.3	136.7±59.9*
**MCP-1 (pg/ml)**	36.8±9.2	80.6±11.9*

Data represent means ± SEM (n = 6–7/group).

*p<.05,

**p<.01,

***p<.001 for *db/db vs. db/+* mice.

### 2–*db/db* mice display increased anxiety-like behaviors and impaired spatial working memory performances

**Figure 1:**
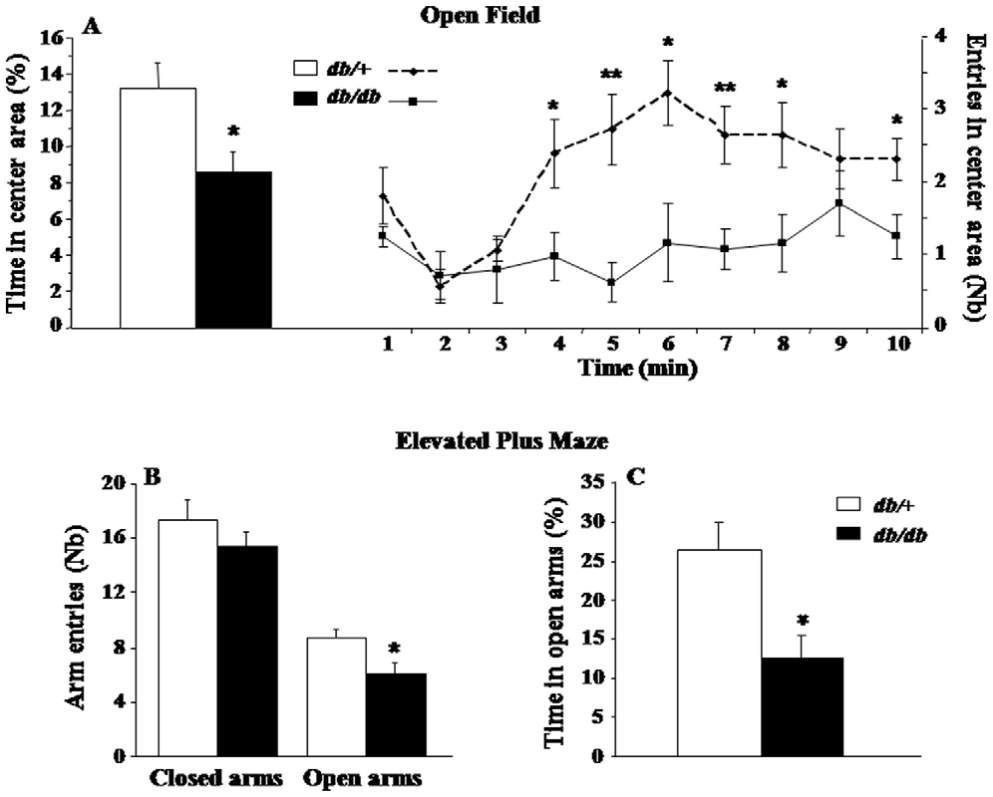
Anxiety-like behaviors of *db/db* and *db/+* mice. (A) Percent of time spent in the center area of the open-field and temporal evolution of the number of entries into this area. (B) Number of entries and (C) percent of time spent into the open arms of the elevated plus-maze. Data represent means ± SEM (n = 6/group). * *p*<.05, ** *p*<.01 for *db/db vs. db/+* mice.

**Figure 2:**
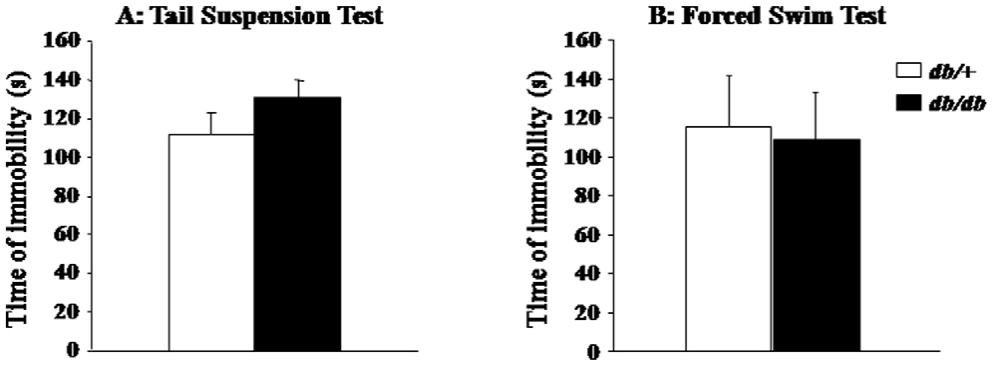
Depressive-like behaviors of *db/db* and *db/+* mice. Immobility time in the (A) tail suspension test and (B) forced swim test. Data represent means ± SEM (n = 7/group).

Anxiety-like behaviors were assessed in the open-field (OF) and elevated plus maze (EPM). When exposed to the OF, *db/db* mice clearly spent less time (F_(1,19)_ = 4.9, p<0.05) and visited less often (F_(1,19)_ = 13.5, p<0.005) the central area than *db/+* mice (Fig. 1A). The detailed time-course analysis of the number of entries performed in this anxiogenic central area revealed a progressive over-time increase in *db/+* mice (time: F_(1,110)_ = 4.4, p<0.0001) whereas central entries remained very few in *db/db* mice throughout the test. Similarly, although all mice equally visited the closed arms of the EPM, *db/db* mice displayed less entries (F_(1,20)_ = 4.5, p<0.05; Fig. 1B) and reduced percent of time spent into the open arms than *db/+* mice (F_(1,20)_ = 6.48, p<0.05; Fig. 1C). Moreover, these behavioral differences were not due to locomotor impairment since *db/db* mice displayed proportionally more entries into the peripheral squares of the OF than *db/+* mice (63.37% *vs.* 55.89%; F_(1,19)_ = 8.7, p<0.01) and both groups exhibited similar number of total arm entries in the EPM (data not shown). Thus, results obtained from the OF and EPM paradigms converge to demonstrate greater anxiety-like behaviors in *db/db* mice compared to their *db/+* counterparts. In contrast, assessment of depressive-like behaviors in the tail suspension test (TST) and forced swim test (FST) reveals similar duration of immobility in both genotypes, suggesting that *db/db* mice did not display greater depressive-like behavior than *db/+* mice (Fig. 2).

**Figure 3:**
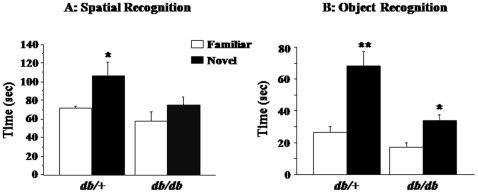
Working memory performances of *db/db* and *db/+* mice. (A) Spatial recognition in the Y-maze expressed as the time spent exploring the novel and the familiar arms. (B) Time spent exploring the novel and the familiar object in the novel object recognition task. In both tasks, measures were assessed over a 5-min test and after 30-min retention. Data represent means ± SEM (n = 7–10/group). * *p*<.05, ** *p*<.01 for *db/db vs. db/+* mice.

We also measured spatial working memory performances of *db/db* and *db/+* mice using a spatial recognition test designed as a hippocampus-dependent task [63], [64]. During the pre-test session of free exploration of two arms out of three in the Y-maze, the number of visits and the time spent in each open arm were similar in both genotypes indicating unaltered spontaneous alternation (data not shown). Spatial memory performances differed across the genotypes when a 30-min retention interval was used, as revealed by main effects of genotype (F_(1,18)_ = 5.6, p<0.05) and arm (F_(1,18)_ = 7.6, p<0.05) (Fig. 3A). Further analysis indicated that *db/+* mice spent more time exploring the novel arm than the familiar arm (p<0.05), whereas *db/db* mice randomly explored the different arms. Consequently, the index of discrimination calculated as the time spent exploring the novel arm divided by the total time exploring the different arms multiplied by 100, was significantly higher than chance level in *db/+* mice (59%, one sample t-test: p<0.05), but not in *db/db* mice (55%, p>0.05) (data not shown). When tested with a short 2-min ITI that corresponds to a minimal mnemonic demand, both *db/db* and *db/+* mice preferentially explored the novel arm compared to familiar arms (F_(1,46)_ = 10.2, p<0.005; 62% and 60% for *db/db* and *db/+* mice, respectively p<0.05) (data not shown). In summary, *db/db* mice displayed normal spatial recognition performances with a short retention interval, but impaired performances once the mnemonic demand increased together with the ITI. These data likely reflect a deficit in hippocampus-dependent working memory rather than motor or motivational disturbances.

To evaluate whether working memory performances were also affected in a non-spatial task, mice were submitted to the novel object recognition (NOR) task used as a hippocampus-independent task [64], [65]. After 30-min retention, all mice explored more the novel object than the familiar one (F_(1,36)_ = 22.5, p<0.0001) (Fig. 3B), although *db/db* mice spent globally less time exploring the objects than *db/+* mice (genotype: F_(1,36)_ = 12.7, p<0.005; object × genotype: F_(1,36)_ = 4.4, p<0.05). Both *db/+* and *db/db* mice were therefore able to discriminate the novel object from the familiar one, as confirmed by the fact that the index of discrimination was statistically different from chance level (50%) whatever the genotype (70.9% and 68.3% respectively; p<0.001). To evaluate whether longer ITI could reveal an impairment of object recognition in *db/db* mice, other mice were tested with a 1-h ITI. Neither *db/db* nor *db/+* mice preferentially explored the novel object over the familiar one with a 1-h ITI, although *db/+* mice still spent more time exploring the objects than *db/db* mice (genotype: F_(1,34)_ = 15.4, p<0.001) (data not shown). In summary, *db/db* mice displayed selective behavioral alterations affecting anxiety-like behavior and spatial working memory.

### 3–Behavioral alterations are associated with peripheral and brain inflammation in *db/db* mice

Table 1 shows peripheral levels of several well-known markers of inflammation. *db/db* mice displayed significantly higher plasma levels of IL-6 (F_(1,10)_ = 6.2, p<0.05) and the chemokine MCP-1 (F_(1,12)_ = 3.0, p<0.05) than *db/+* mice. Plasma levels of IL-1β and TNF-α were low and similar in both genotypes, whereas no IFN-γ was detected whatever the group.

**Figure 4:**
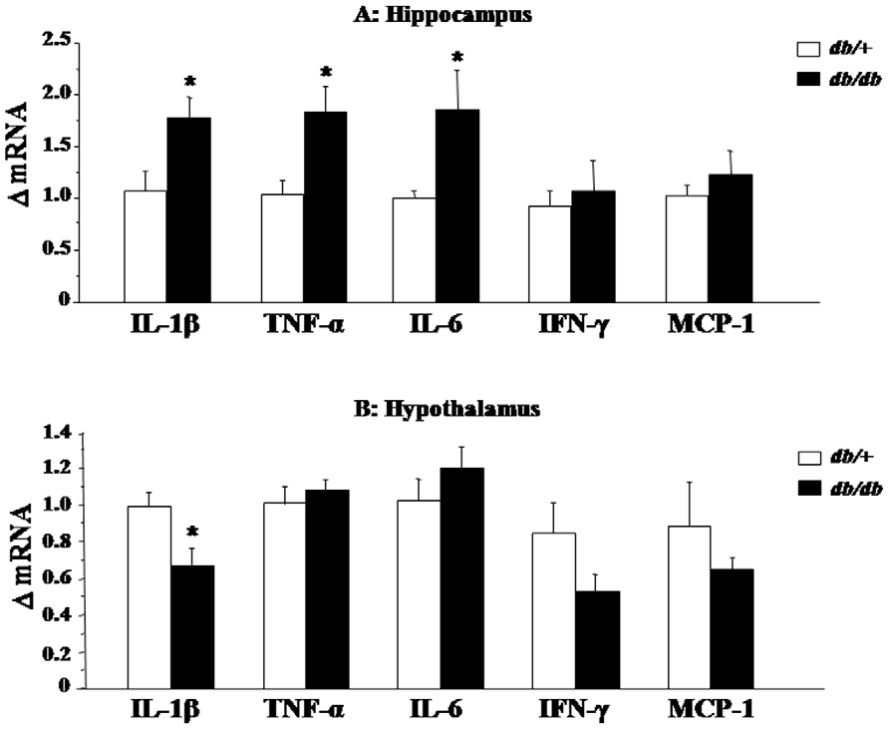
mRNA expression levels of cytokines in the hippocampus and hypothalamus of *db/db* and *db/+* mice. Relative fold changes in the levels of (A) hippocampal and (B) hypothalamic IL-1β, TNF-α, IL-6, IFN-γ and MCP-1 mRNA expression, as calculated in relation to the averaged value for control saline group. IL-1β, interleukin-1β; TNF-α, tumor necrosis factor-α; IFN-γ, interferon-γ; MCP-1, monocyte chemotactic protein-1. Data represent means ± SEM (n = 6/group). * *p*<.05 for *db/db vs. db/+* mice.

Cytokine mRNA expression was measured in the hippocampus, a key brain areas for the control of spatial memory and anxiety-like behaviors [43], [44]. Concomitantly, the hypothalamus was used as a control structure involved in physiological homeostasis, but not in the control of anxiety-like and cognitive behaviors [44], [46]. IFN-γ and MCP-1 mRNA expression levels were similar regardless the genotype and the area. Hippocampus mRNA expression of IL-1*β* (F_(1,9)_ = 6.8, p<0.05), TNF-α (F_(1,10)_ = 7.4, p<0.05) and IL-6 (F_(1,8)_ = 7.1, p<0.05) was significantly higher in *db/db* mice than in their *db/+* counterparts (Fig. 4A). In contrast, hypothalamus TNF-α and IL-6 mRNA expression was similar in both genotypes whereas *db/db* mice displayed lower levels of IL-1*β* mRNA expression than *db/+* mice (F_(1,9)_ = 7.6, p<0.05; Fig. 4B).

**Figure 5:**
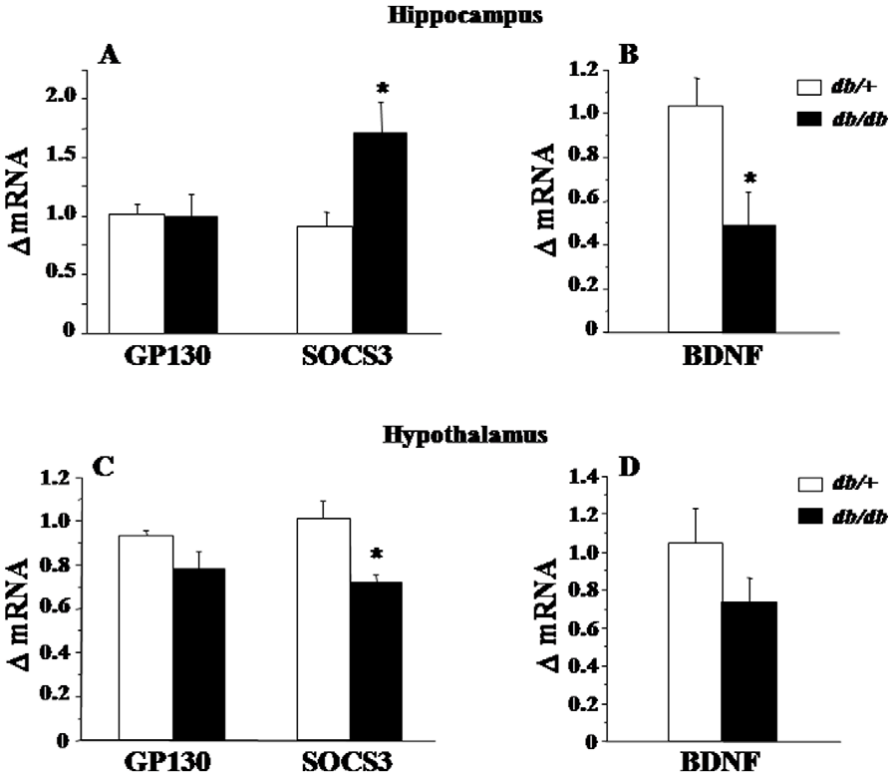
mRNA expression levels of GP130, SOCS3 and BDNF in the hippocampus and hypothalamus. Relative fold changes in the levels of (A–B) hippocampal and (C–D) hypothalamic GP130, SOCS3 and BDNF mRNA expression, as calculated in relation to the averaged value for control saline group. GP130, glycoprotein 130; SOCS3, suppressor of cytokine signaling-3; BDNF, brain-derived neurotrophic factor. Data represent means ± SEM (n = 6/group). * *p*<.05 for *db/db vs. db/+* mice.

To further understand the functional consequences of increased expression of cytokines, we next measured mRNA expression of one of the key element of the IL-6 receptor complex, the glycoprotein 130 (GP130), IL-6 being the only cytokine increased at the periphery and in the hippocampus of *db/db* mice. Additionally, we measured the mRNA expression of cytokine signaling-3 (SOCS3), a classical indicator of cytokine signaling pathway activation [66]. GP130 mRNA expression was stable across the brain area and the genotype (Fig. 5A, 5C). On the contrary, *db/db* mice displayed increased levels of SOCS3 mRNA expression in the hippocampus (F_(1,9)_ = 6.7, p<0.05; Fig. 5A), together with a reduced expression in the hypothalamus (F_(1,9)_ = 12.1, p<0.01; Fig. 5C). Alterations displayed by *db/db* mice in anxiety-like behaviors and spatial working memory were therefore associated with hippocampal, but not hypothalamic, low-grade inflammation.

Finally, we measured the mRNA expression of BDNF, a potent neuroprotective growth factor regulated by cytokines [48]–[50] and well-known to participate in mood regulation and memory function [45], [47]. BDNF was significantly reduced in the hippocampus of *db/db* mice compared to *db/+* mice (F_(1,9)_ = 7.8, p<0.05; Fig. 5B), whereas no significant differences were observed between both groups in the hypothalamus (Fig. 5D).

## Discussion

Our study allows for the first time to directly relate in *db/db* mice increased anxiety-like behaviors and impaired spatial memory performances with activation of specific inflammatory pathways within the hippocampus, a key brain area for the control of emotional and cognitive behaviors.

Most clinical studies investigating the link between MetS and mood disorders report a positive association [6], [10], [15]. Although measuring mood in rodents could appear limiting, the development of consistent and reliable behavioral tests modeling different core symptoms of anxiety and depression rather than the entire syndromes has provided very useful tools to study their respective pathophysiology [67]. Here, data obtained in different experimental paradigms converge to indicate that *db/db* mice display increased anxiety-like behaviors, as previously suggested [62], but not depressive-like behaviors. Increased immobility in the FST has been reported in leptin-deficient (*ob/ob*) mice when experimental conditions much more stressful than ours are used (longer exposure to the FST and for 3 consecutive days) [68]. Of note, depressive-like behaviors in the FST and/or TST mostly increase under challenging conditions such as stress exposure [69] or immune stimulation [24]–[26], [70]. The possibility that *db/db* mice would display greater depressive-like behaviors in such conditions is currently under study. While this may be, the current study clearly shows that *db/db* mice displayed increased anxiety-like, but not depressive-like, behaviors in basal conditions.

Clinical studies have reported that MetS adversely impairs cognition, although not all cognitive domains are equally affected [4], [5], [11]. Our study further extends these findings by demonstrating task-specific cognitive impairments in *db/db* mice using two working memory paradigms (the Y-maze and NOR). After 30 minutes of retention, *db/db* mice exhibited spatial working memory impairment in the Y-maze while their performances were unaltered in NOR memory task. Moreover, the spatial deficit in *db/db* mice is not due to motor and/or motivational problems as their spatial performance was intact with a shorter retention interval (2-min). Intact performances of *db/db* mice in the NOR task disagree with recently published data showing NOR impairment in these mice with the same 30-min ITI [61]. However, two important differences between their experimental protocol and ours can likely explain this apparent discrepancy. First, they used C57BL/6J mice as controls instead of *db/+* mice (as we used), which have similar genetic background (C57BL/6J×DBA/2J) and perinatal environment as their littermates *db/db* mice. Interestingly, we recently showed differences in the duration of object exploration between C57BL/6J and *db/+* mice (data not shown) that can likely be explained by their different genetic background and/or different perinatal environment. Second and more importantly, they exposed *db/db* mice only once to the testing cage before the NOR training and test, whereas in our protocol mice were extensively habituated to the testing cage (15 min per day during 8 days). According to the fact that the level of habituation to the experimental context influences the emotional arousal and performances during NOR [71], [72] and that *db/db* mice show greater anxiety-like behaviors than controls, it is likely that testing *db/db* mice in higher stressful conditions than ours may interfere with their NOR performances.

In parallel to behavioral alterations, we report neurobiological changes in *db/db* mice affecting molecules well-known to participate in emotional and cognitive behaviors, particularly cytokines [21], [22]. Expression level of IL-6, TNFα and IL-1β mRNAs was selectively increased by ∼80% in the hippocampus, but not the hypothalamus, of *db/db* mice. Although we only measured cytokine transcripts, these changes are likely to be reflected in changes of protein levels contributing to downstream neurobiological and behavioral modulations [73]. This assumption is supported by the concomitant increase of hippocampal SOCS3 mRNA expression that is classically used as an indicator of cytokine signaling pathway activation, mainly IL-6 [66]. In contrast, IL-1β mRNA expression is significantly reduced in the hypothalamus of *db/db* mice. This can be linked to the increased food intake of these mice as mounting evidence indicates the anorectic effect of enhanced hypothalamic expression of IL-1 [21], [74], including in response to leptin [75]. Of note, this result is particularly relevant to better understand the role of brain cytokines in the impairment of food intake in *db/db* mice, although this question has not been directly addressed in the present study. As reported for cytokines, BNDF is also well-known to contribute to mood regulation and memory function, particularly in the hippocampus [45], [47] where its expression is negatively regulated by cytokines [48], [49]. Accordingly, the increase of proinflammatory cytokine expression we found in the hippocampus of *db/db* mice is accompanied by ∼60% decrease of hippocampal, but not hypothalamic, BDNF mRNA expression. This change is likely reflected in changes of functional protein levels since significant correlations have been reported between both BDNF mRNA expression and protein levels [76], [77].

These neurobiological changes are observed in the hippocampus, but not in the hypothalamus. This is particularly relevant in light of the behavioral alterations of *db/db* mice. Indeed, we found that *db/db* mice are impaired in spatial, but not in object, recognition tasks for a similar delay and considerable literature shows that spatial memory, but not object memory, requires normal hippocampal functioning [65], [78]. This interpretation agrees with data reporting that *db/db* mice are impaired in the hidden-platform version of the Morris water-maze test (a hippocampus-dependent situation), but not under visible-platform conditions (a hippocampus-independent situation) [59]–[61]. Moreover, the hippocampus is also involved in anxiety-related behaviors [43]. Therefore the higher level of anxiety-like behaviors we found in *db/db* mice (see also Stranahan et al. [62]) could also be linked to the neurobiological changes we found in the hippocampus of these mice, although further studies are needed to measure potential neurobiological changes in other brain areas well-known to participate in controlling emotional behavior, particularly the amygdala complex (for instance see [79]).

All these data support a key role for the hippocampal changes (increased cytokines, decreased BDNF) in the cognitive and emotional alterations displayed by *db/db* mice. Interestingly, chronic overexpression of brain IL-6 or hippocampal IL-1β in transgenic mice impairs learning [37], [41]. These findings concur with several reports highlighting the relationship between neuroinflammation, alterations of hippocampal synaptic plasticity and impaired cognitive performances [38], [39], [51]. Interestingly, different animal studies report that increased levels of hippocampal IL-6 interfere with local long-term potentiation, neurogenesis and synaptic plasticity [40]–[42], whereas blocking hippocampal IL-6 intracellular pathway prevents cytokine-induced alterations of synaptic activity and spatial learning [80]. In humans, circulating IL-6 concentrations have been shown to covary inversely with hippocampal grey matter volume [36] and cognitive performances [32]. Chronic proinflammatory cytokines have also been shown to produce detrimental effects on mood [22]–[26]. Consistent clinical data report a significant correlation between elevated circulating levels of inflammatory markers, particularly IL-6, and development of anxiety symptoms [33], [81], including in obese patients [53], [54]. Likewise, experimental studies using IL-6-deficient mice [30] or rats bred for extremes in anxiety-related behavior [82] support a role of IL-6 in anxiety-like behavior. Concerning BDNF, decreased level in the hippocampus is associated with impaired synaptic plasticity, cognitive performances [45] and mood-related behaviors [47]. Interestingly, IL-1β modulates memory-induced increase in hippocampal BDNF mRNA expression and signal transduction [21], [48], [50]. Accordingly, elevated cytokine levels observed in the hippocampus of *db/db* mice may contribute to decrease BDNF level and consequently to impair anxiety-like behaviors and spatial cognitive performances.

Beyond elevated cytokine levels and related neurobiological changes, *db/db* mice also displayed elevated plasma levels of leptin, insulin and corticosterone. In addition to their metabolic properties, leptin and insulin can modulate behavior by acting within the brain [83], [84]. Consequently, impaired leptin and/or insulin signaling activation, as observed in *db/db* mice, has been proposed as potential link between behavioral and metabolic disorders. Although this assumption cannot be totally excluded based on the present data, several lines of evidence suggest that leptin does not play a main role in mediating behavioral alterations of *db/db* mice. Indeed, cognitive impairment displayed by *db/db* mice can be improved by normalization of corticosterone levels, without restoring impaired leptin signaling pathway [61], [62]. Moreover, caloric restriction and running enhance exploratory behavior in *db/db* mice, without changing serum leptin levels [62]. Similarly, insulin unlikely contributes to the behavioral alterations reported in *db/db* mice. Indeed, brain concentrations of glucose and insulin are similar in both *db/db* and *db/+* mice and remained unchanged after normalization of peripheral hyperinsulinemia [61]. Moreover, caloric restriction and running completely reversed hyperglycemia in *db/db* mice, without reducing anxiety-like behavior in the open-field [62]. Likewise, normalizing hyperglycemia in insulin-deficient rats does not reverse spatial cognitive impairment [85]. These data match those showing that the increased risk of cognitive dysfunction reported in MetS patients is independent from the presence of diabetes [4]. Additionally, it was recently suggested that elevated corticosterone contributes to cognitive alterations in *db/db* mice by acting within the hippocampus [61]. Normalizing plasma corticosterone levels reverses alterations of hippocampal plasticity and improves hippocampus-dependent memory [61]. However, these findings do not preclude the involvement of other factors, namely proinflammatory cytokines, in cognitive and emotional alterations. As both the inflammatory system and the hypothalamic-pituitary-adrenal axis have been found tightly interrelated [86], it would be of interest to assess in *db/db* mice whether normalizing plasma corticosterone also normalizes hippocampal proinflammatory cytokines. Additionally, this study should be broadened to other brain areas, particularly the amygdala complex, since 1) it plays a key role in controlling emotional behavior, particularly anxiety [79] and 2) it can be targeted by both corticosterone and cytokines [87]. Based on all these converging data, it can reasonably be proposed that the behavioral profile displayed by *db/db* mice likely results from the concerted interactions of cytokines and glucocorticoids with central processes involving the hippocampus and controlling cognition and emotionality.

In conclusion, although only correlative and with the limitation inherent to the measurement of neurobehavioral reactivity in rodents, the present results strongly point to hippocampal inflammation as important contributing factors to the pathophysiology of neuropsychiatric complications associated with MetS. These findings may prove valuable for introducing novel approaches to treatment, alongside currently used non-pharmacologic and pharmacologic interventions.

## Materials and Methods

### Animals

All animal experiments were conducted according to the INRA Quality Reference System, and to relevant French (Directive 87/148, Ministère de l'Agriculture et de la Pêche) and international (Directive 86/609, November 24^th^ 1986, European Community) legislation. They adhered to protocols approved by Région Aquitaine Veterinary Services (Direction Départementale de la Protection des Animaux, approval ID: A33-063-920). Every effort was made to minimize suffering and the number of animal used. Male *db/db* (C57BLKS/J-*lepr^db^/lepr^db^*; n = 40) and *db/+* (C57BLKS/J-*lepr^db^/+*; n = 40*)* mice obtained from Charles River Laboratories (France) were housed individually under a normal 12-hour light:dark cycle with food and water available *ad libitum*. Mice were handled daily for 1 week before the experiment onset to minimize stress reactions to manipulation. They were 8 to 10 weeks-old at the time of behavioral assessments.

### Behavioral measurements

Experiments were performed in the morning under conditions of dim light and low noise. Behavior was videotaped and scored using “The Observer Basic” software (Noldus, Netherlands). Each mouse was submitted to a maximum of two different behavioral tests, with a 1-week interval between tests. All testing equipment was thoroughly cleaned between each session.

#### Open-field (OF)

Mice were exposed to an unknown square area (40×40 cm) from which escape is prevented by surrounding walls (16 cm high). The apparatus was virtually divided into 4 central squares defined as the central area and 12 squares along the walls that are defined as the periphery. Each mouse was placed in the central area and allowed to freely explore the OF for 10 min. Parameters recorded to evaluate anxiety-like behavior were the number of entries and the percent of time spent in the central area [88].

#### Elevated Plus Maze (EPM)

The EPM was a plus shaped acryl maze with two opposing open arms (30×8 cm) and two opposing closed arms (30×8×15 cm) connected by a central platform (8×8 cm) and elevated 120 cm above the floor. Each mouse was placed in the center of the maze facing an open arm and the number of arm entries, as well as the percent of time spent in open arms, was assessed during a 5-min period. An entry was scored as such only when the mouse placed all four limbs into any given arm. A reduction of the percent of time spent and number of entries into the open arms is considered as an anxiety-like index, independent of locomotor activity [88].

#### Tail Suspension Test (TST)

This standardized test of depressive-like behavior was carried out as previously described [24], [70]. Briefly, an adhesive tape was fixed to the mouse tail and hooked to a horizontal ring stand bar placed 30 cm above the floor. The test was conducted for a 6-min period in a visually isolated area. Mice demonstrated several escape attempts interspersed with immobility periods during which they hung passively and completely motionless. Depressive-like behavior was inferred from increased duration of immobility.

#### Forced Swim Test (FST)

This standardized test of depressive-like behavior was essentially conducted as previously described [24], [70]. Briefly, each mouse was placed individually in a cylinder (16×31 cm) containing warm water (25±1°C) to avoid temperature-related stress response. Mice were tested during a 6-min period. Immobility time was determined by the time a mouse stopped struggling and moved only slowly to remain floating in the water, keeping its head above water. Increased duration of immobility has been proposed to reflect a state of helplessness that is reduced by antidepressants.

#### Y-maze

Spontaneous spatial recognition in the Y-maze was used as a hippocampal-dependent test as previously described [63], [64]. The apparatus was a Y-shaped acryl maze with three identical arms (34×8×14 cm). Corncob litter covered the floor and was mixed between each trial in order to remove olfactory cues. Visual cues were placed in the testing room and kept constant during the whole test. Discrimination of novelty versus familiarity was based on the different aspects of the environment that the mouse can perceive from each arm of the Y-maze. In the first trial of the test (acquisition), one arm was closed with a door and mice were allowed to freely visit the two other arms for 5 min. After a 30-min inter-trial interval (ITI), mice were again placed in the start arm for the second trial (retrieval) and allowed free access to all three arms for 5 min. Start and closed arms were randomly assigned to each mouse. Arm entries were defined as all four paws entering the arm. Preference for novelty was also measured using a short 2-min ITI between acquisition and retrieval in order to control for potential motivational disturbances [63], [64]. Analyses were based on the time spent exploring the novel and the familiar arms during the second trial. An index of discrimination between novel and familiar arms was calculated as the ratio of the [time spent in the novel arm/(time spent in the novel + adjacent arms)]×100. An index of discrimination significantly higher than chance level (50%) indicates therefore that mice indeed recognize the novel arm.

#### Novel object recognition (NOR)

This task is a free exploration paradigm allowing mice to explore objects in a non-threatening and familiar environment. It was used as a hippocampal-independent task [64], [65]. Mice were first acclimatized in an opaque acryl cage (43×28×19 cm) for 15 min per day during the week before training. The floor was covered with corncob litter that was mixed between each trial in order to remove olfactory cues. On the ninth day, mice were placed in the experimental cage with two identical objects during a 10-min training session. Three sets of objects with different shapes and colors were used for discrimination. Each object was chosen on the basis of a preliminary study examining the preferences of a separate group of mice (data not shown) and was heavy enough not to be displaced by a mouse. After a 30-min or 1-hour ITI, one of the familiar objects was replaced by a novel object, with a different shape and color, to test for memory retention. During the 5-min test, exploration of each object was defined as sniffing or touching the object with the nose and/or forepaws. Analyses were based on the time spent exploring the novel and the familiar objects. An index of discrimination was calculated as the ratio of the [time spent exploring the novel object/(total time spent exploring both objects)]×100.

### Biochemical measurements

After completion of the behavioral experiments, some *db/db* and *db/+* mice were euthanized by CO_2_ inhalation (10–12 weeks-old at the time of sacrifice). Blood samples were immediately collected via cardiac puncture into EDTA (10%)-coated chilled tubes. After centrifugation (10 min, 3000 *g*, 4°C), aliquots of plasma were stored at −80°C. Mice were perfused with chilled PBS via the ascending aorta to remove all traces of blood from tissues. Brains were rapidly extracted from the skulls and carefully dissected. The hippocampus and the hypothalamus were immediately collected, dried frozen and stored until assaying.

#### Hormones and cytokines assay

As previously described [24], [35], plasma leptin, insulin, resistin, monocyte chemotactic protein-1 (MCP-1), IL-1β, IL-6, TNF-α and IFN-γ were measured with the mouse adipokine and cytokine LINCOplex kits (Linco Research, Inc., St. Charles, MO, USA) following the manufacturer's instructions. Plasma corticosterone concentrations were measured using a commercial RIA Kit (Diasorin, Antony, France). Plasma glucose levels were measured using a One Touch Ultra glucometer per the manufacturer's instructions. All samples were run in duplicate.

#### Reverse transcription and real-time RT-PCR

Total RNA was extracted from the hippocampus and hypothalamus using a RNeasy Mini Kit (Qiagen) and reverse-transcribed as previously described [25], [26], [35], [64]. Real-time RT-PCR was performed on an ABI Prism 7700 using Taqman gene expression assays for sequence-specific primers purchased from Applied Biosytems (Foster City, CA). Reactions were performed in duplicate according to manufacturer instructions as previously described [25], [26], [35]. Relative expression levels were calculated according to the methods of Livak and Schmittgen [89] and plotted as fold change relative to the appropriate control condition.

### Statistical analysis

Results are presented as mean ± SEM and were analyzed using a one-way (genotype) or a two-way (genotype×arm; genotype×object) ANOVA followed by a *post-hoc* pair wise multiple comparison procedure using the Fischer's LSD method, if the interaction was significant.
